# Fabrication and characteristics of multifunctional hydrogel dressings using dopamine modified hyaluronic acid and phenylboronic acid modified chitosan

**DOI:** 10.3389/fchem.2024.1402870

**Published:** 2024-05-22

**Authors:** Yanting Han, Jing Cao, Man Li, Peng Ding, Yujie Yang, Oseweuba Valentine Okoro, Yanfang Sun, Guohua Jiang, Amin Shavandi, Lei Nie

**Affiliations:** ^1^ College of Life Sciences, Xinyang Normal University, Xinyang, China; ^2^ Université libre de Bruxelles (ULB), École polytechnique de Bruxelles—3BIO-BioMatter, Brussels, Belgium; ^3^ College of Life Sciences and Medicine, Zhejiang Sci-Tech University, Hangzhou, China; ^4^ School of Materials Science and Engineering, Zhejiang Sci-Tech University, Hangzhou, China; ^5^ International Scientific and Technological Cooperation Base of Intelligent Biomaterials and Functional Fibers, Zhejiang Sci-Tech University, Hangzhou, China

**Keywords:** hyaluronic acid, chitosan, hydrogels, cytocompatibility, wound dressing

## Abstract

The healing of damaged skin is a complex and dynamic process, and the multi-functional hydrogel dressings could promote skin tissue healing. This study, therefore, explored the development of a composite multifunctional hydrogel (HDCP) by incorporating the dopamine modified hyaluronic acid (HA-DA) and phenylboronic acid modified chitosan (CS-PBA) crosslinked using boric acid ester bonds. The integration of HA-DA and CS-PBA could be confirmed using the Fourier transform infrared spectrometer and ^1^H nuclear magnetic resonance analyses. The fabricated HDCP hydrogels exhibited porous structure, elastic solid behavior, shear-thinning, and adhesion properties. Furthermore, the HDCP hydrogels exhibited antibacterial efficacy against Gram-negative *Escherichia coli* (*E. coli*) and Gram-positive *Staphylococcus aureus* (*S. aureus*). Subsequently, the cytocompatibility of the HDCP hydrogels was verified through CCK-8 assay and fluorescent image analysis following co-cultivation with NIH-3T3 cells. This research presents an innovative multifunctional hydrogel that holds promise as a wound dressing for various applications within the realm of wound healing.

## 1 Introduction

The skin serves to prevent the invasion of microorganisms and dehydration. Although the human skin has considerable self-regeneration capabilities, it is vulnerable to skin defects that cannot spontaneously heal in extreme situations (Graça et al., 2020; Tavakoli and Klar, 2020). The skin injury caused by cuts, tears, or punctures resulting from external stimulation or trauma, such injuries can inflict permanent pain and lead to long-term psychological distress in patients (Cai et al., 2019; Tang et al., 2021a; Cao et al., 2021; Ding et al., 2021). The wound healing process is intricate and involves overlapping stages of hemostasis, inflammation, proliferation, and remodeling (Liang et al., 2021a). The appropriate wound dressing is necessary for the restorative process of wound healing. To promote wound repairs, dressings of different types, such as membranes (semipermeable), gauze, film, hydrocolloids, hydrogels, *etc.*, have been investigated (Han et al., 2017; He et al., 2020; Li et al., 2020). Among these, hydrogels are recognized as the preferred candidates for the fabrication of wound dressings due to their porous structure resembling the extracellular matrix, customizable mechanical properties, superb tissue compatibility, favorable hydrophilicity, and hygroscopicity properties (Ahmed, 2015). Besides, the properties of hydrogels are beneficial in that they can absorb exudates or blood, thus providing an environment that is moist and clean, allowing for the exchange of oxygen and water permeability in areas affected by skin defects. This supports wound healing and helps alleviate the pain experienced by patients (Nie et al., 2020; Tang et al., 2021b; Jin et al., 2022; Zou et al., 2022; Zeng et al., 2023). Diverse biopolymers like chitosan, collagen, hyaluronic acid, and others have been utilized to create hydrogels for wound dressings. The multifunctional hydrogels could be fabricated using crosslinking approaches, such as borate ester bonds and Schiff base bonds (Rasool et al., 2019; Shavandi et al., 2020; Dhand et al., 2021; Jafari et al., 2021; Mirzaei et al., 2021). Earlier studies have shown that the boric acid ester bonds established between diols and boronic acid offer numerous benefits, including an easy preparation process, mild reaction conditions, and effective tissue-adhesive properties (Cambre and Sumerlin, 2011; Kotsuchibashi et al., 2013; Hong et al., 2020; Yang et al., 2022). The wound dressing incorporating boric acid ester bonds demonstrates a dynamic recovery feature, responding to breakage and formation while being susceptible to destruction in low pH and low glucose concentrations, making it responsive to the microenvironment of diabetic ulcers. Furthermore, the hydrogel, crosslinked through boric acid ester bonds, exhibits loading and release of bioactive agents, thereby enhancing their utilization. Therefore, wound dressings based on the crosslinking of boric acid ester bonds need to be further explored.

Among common biopolymers, hyaluronic acid (HA) is a high molecular weight polymer compound with biocompatibility, biodegradability, and hydrogel characteristics. These attributes render it well-suited for applications in wound treatment (Grégoire et al., 2023; Nie et al., 2023). As a extracellular component, it plays a vital role in the skin wound repair process. Previous studies have demonstrated that HA can facilitate cell signal transduction, enhance the proliferation and differentiation of endothelial cells, support cell migration, angiogenesis, and modulate inflammation in the course of wound healing. This ultimately contributes to tissue regeneration. Due to these characteristics, HA stands out as a compelling candidate for use as a tissue adhesive (Zhou et al., 2020; Tang et al., 2021c). However, the main drawback of HA is its insufficient adhesive performance to close the wound site. Studies have indicated that hydrogels based on polydopamine or containing catechol frequently exhibit strong wet tissue-adhesive capabilities. This phenomenon can be attributed to chemical crosslinking and physical bonding occurring between the polydopamine or catechol groups and soft tissues (Liang et al., 2019; Cui et al., 2022). Therefore, in this research, dopamine was grafted onto HA to compensate for the insufficient adhesion of HA, which also gives the wound dressing enhanced adhesiveness, which is beneficial for wound repair.

The natural polysaccharide of chitosan (CS) is known for its biocompatibility, bioactivity, and biodegradability. It exhibits unique antioxidative, antibacterial, anti-inflammatory, and hemostatic characteristics, rendering it especially beneficial for use in wound treatment applications (Deng et al., 2021; Deng et al., 2022; Liu et al., 2022; Nie et al., 2022; Sun et al., 2022; Wu et al., 2022; Ding et al., 2024; Guo et al., 2024). The antibacterial action of chitosan primarily relies on the presence of amino groups along its linear molecular chain. These amino groups have the ability to bind with acidic molecules, acquiring a positive charge. As a result, chitosan can interact efficiently with the negatively charged regions of bacterial proteins, resulting in the deactivation of bacteria. Chitosan demonstrates extensive antibacterial effectiveness against Gram-positive and Gram-negative bacteria, attributed to its abundant hydroxyl and amino groups capable of forming numerous hydrogen bonds. Consequently, it possesses strong chelating adsorption ability, effectively adsorbing charged substances and finding applications in various fields. However, limitations arise when the pH of chitosan exceeds 6, leading to expansion upon water absorption, poor tensile mechanical properties, rapid degradation, and challenging dissolution. These factors impose some constraints on its application.

**SCHEME 1:**
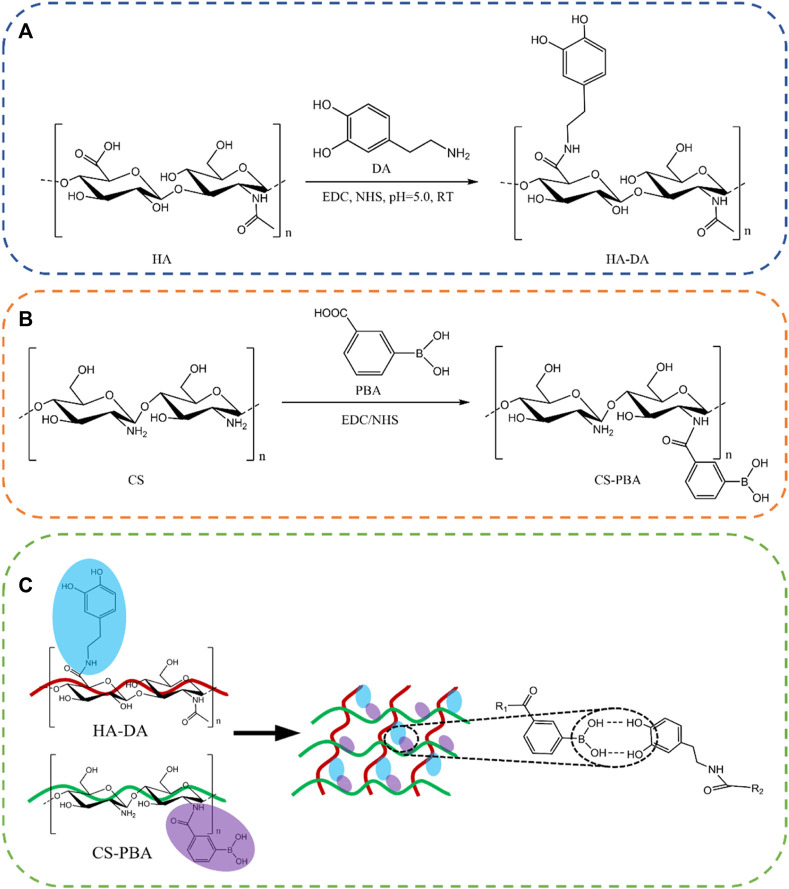
Reaction structure diagram of HA-DA **(A)** and CS-PBA **(B)**. **(C)** Schematic diagram of HA-DA/CS-PBA hydrogel.

To address above limitations, chitosan is frequently combined with other polymer materials to create composite hydrogels, mitigating the mentioned issues (Ding et al., 2024; Guo et al., 2024). The modification of chitosan with the grafting of carboxymethyl and quaternary ammonium salt groups enhanced the solubility of chitosan and its antibacterial properties (Guo et al., 2024). Alternatively, additional biochemical modifications can be employed to enhance its water solubility and overall performance (Ding et al., 2020). To further increase the solubility and cytocompatibility of chitosan, chitosan was modified by phenylboronic acid to obtain products of phenylboronic acid modified chitosan (CS-PBA) in this paper. At the same time, HA was modified using dopamine to produce dopamine modified hyaluronic acid (HA-DA). Based on the boric acid ester bonds between synthesized CS-PBA and HA-DA, the multifunctional composite hydrogel (HDCP) was fabricated (Scheme 1).

## 2 Materials and methods

### 2.1 Chemicals

Chitosan (CS), with a deacetylation degree of 80%–95% and viscosity ranging from 50 to 800 cP, was sourced from Ron reagent in Shanghai, China. Dopamine hydrochloride (DA) with a purity of 98%, 3-carboxyphenylboronic acid, N-(3-dimethylaminopropyl)-N′-ethylcarbodiimide hydrochloride (EDC), N-hydroxy succinimide (NHS), and 2-Morpholinoethanesulfonic acid (MES) were obtained from Aladdin Biochemical Technology Co., Ltd in Shanghai, China. Hyaluronic acid (HA) was acquired from Macklin Biochemical Technology Co., Ltd in Shanghai, China. Fetal bovine serum, trypsin, penicillin, and streptomycin were purchased from Wuhan Huashun Biotechnology Co., Ltd in Wuhan, China. All other solvents and chemicals utilized in this study were of analytical grade and employed without further purification.

### 2.2 Synthesis of dopamine modified hyaluronic acid (HA-DA)

The preparation of HA-DA followed a previously reported method with certain modifications. (Cui et al., 2022). Briefly, 1.0 g of CS was dispersed in 100 mL of deionized (DI) water. Subsequently, 575 mg of EDC and 345 mg of NHS were introduced and stirred for 30 min. Following this, 569 mg of dopamine hydrochloride was added to the mixture, maintaining the pH at 5.0 using MES for 3 h. The reaction was conducted at 37°C for 24 h under a nitrogen atmosphere. Following the reaction, the mixture underwent dialysis under acidic conditions for 3 days to remove unreacted molecules. The resulting product, HA-DA, was obtained through freeze-drying and then stored at −20°C for subsequent use.

### 2.3 Synthesis of phenylboronic acid modified chitosan (CS-PBA)

Initially, 2 g of chitosan (CS) was dissolved in a 1% acetic acid solution. Once complete dissolution was achieved, the pH of the solution was adjusted to 5.5 using 50% MES. Subsequently, 30 mL of N-N dimethyl diamide solution (20% wt), 1.5 g of 3-aminobenzenboric acid, 2.079 g of EDC, and 1.248 g of NHS catalyst were added and stirred for 30 min. Following thorough mixing, the mixture was stirred at room temperature (RT) to ensure sufficient reaction. Finally, the mixture underwent dialysis (MWCO = 10,000 Da) with deionized (DI) water for 3 days. After the dialysis process, the resulting products were freeze-dried and designated as CS-PBA.

### 2.4 Preparation of HDCP composite hydrogels

Initially, the HA-DA polymer was dissolved in 3% PVA water to create solutions of 1 wt% (w/v), 1.5 wt% (w/v), and 3 wt% (w/v) HA-DA, respectively. Simultaneously, the CS-PBA polymer was dissolved in DI water to generate a 3 wt% (w/v) CS-PBA solution. Subsequently, HDCP composite hydrogels were formed by combining the CS-PBA solution and HA-DA solutions in a 1:1 (v/v) ratio at 25 °C. In this study, HDCP hydrogels were prepared using 1 wt% (w/v), 1.5 wt% (w/v), and 3 wt% (w/v) HA-DA solutions, denoted as HDCP-1, HDCP-1.5, and HDCP-3, respectively. According to the ^1^H NMR results, the molar ratio of phenylboronic acid and dopamine in HDCP-1, HDCP-1.5, and HDCP-3 were 1.25, 0.625, and 0.417, respectively.

### 2.5 ^1^H nuclear magnetic resonance (^1^H NMR)

The synthesized polymers (HA, CS, HA-DA, CS-PBA) were dissolved in D2O and subjected to analysis using a nuclear magnetic resonance spectrometer (^1^H NMR, 600 MHz, NMR spectrometer, JNM ECZ600R/S3). Additionally, the effective crosslinking between HA-DA and CS-PBA in HDCP composite hydrogels was validated through ^1^H NMR (Lin et al., 2020; Jabrail et al., 2023).

### 2.6 Fourier transform infrared spectroscopy (FT-IR)

The Fourier transform infrared spectrometer (FT-IR, ThermoFisher) was employed to analyze the functional groups present in HA-DA and CS-PBA polymers and examine potential chemical interactions within HDCP composite hydrogels. FT-IR spectra were collected within the range of 400–4,000 cm^-1^.

### 2.7 Scanning electron microscopy (SEM)

The morphologies and microstructures of the fabricated HDCP hydrogels were examined using a cold field emission scanning electron microscope (SEM, Hitachi, Regulus8220). The freeze-dried samples were frozen in liquid nitrogen for 5 min and then sliced into thin sections. Prior to analysis, a platinum layer was applied to the sample surfaces and maintained for 120 s.

### 2.8 Rheological measurement

Dynamic rheological testing at room temperature was executed using a modular compact rheometer (TA Instruments, New Castle, DE) equipped with a circular spline having a 25 mm diameter. An oscillating strain sweep experiment was employed to determine the linear viscoelastic region of the hydrogel. The strain sweep spanned from 0.1% to 1,000% at a frequency of 1 Hz. Following this, a frequency sweep test was conducted on the hydrogel spline under constant conditions of γ = 0.5%, covering frequencies from 0.01 rad/s to 500 rad/s. Additionally, the shear flow properties of the hydrogels were characterized through oscillation frequency experiments performed at 25°C. These experiments involved maintaining a constant strain of 1% while altering the shear rate from 0.1 rad/s to 100 rad/s.

### 2.9 Swelling rate

The freeze-dried HDCP hydrogels were measured, and the initial mass (*W*_*A*_) in grams was recorded. Subsequently, the hydrogels were immersed in water and removed at specific times. Following retrieval, the hydrogels were reweighed (*W*_*B*_), and the mass in grams was documented, with excess water removed using filter paper from the hydrogel surface. The swelling rate (SR) of the hydrogels was calculated using Eq. 1 as follows:

$$\text{SR }\left(\%\right)=\frac{W_{B}-W_{A}}{W_{A}}\times 100\%$$ (1)

### 2.10 Adhesive performance

The adhesive characteristics of HDCP hydrogels were evaluated by examining their performance upon direct application to finger, arm, and plastic surfaces. The HDCP hydrogel specimen was attached to the surface of substrates with a little pressure for 1 min, and the photos were recorded, and the adhesive performance was confirmed by keeping the hydrogel attached for 10 min.

### 2.11 Antioxidation analysis

Stable 1,1-diphenyl-2-picrylhydrazyls (DPPH) free radicals were employed to assess the antioxidant properties of HDCP hydrogels. In summary, hydrogels were introduced into the DPPH solution, followed by stirring and incubation in darkness for 30 min. The wavelength of DPPH in the mixture was then determined using a UV-Vis spectrophotometer (Lambda950) over a range of 200 nm–800 nm. The absorbance value at 517 nm was measured, and the scavenging ratio was calculated using the following Eq. 2:

$$\text{DPPH scavenging }\left(\%\right)=\frac{A_{0}-A_{1}}{A_{0}}\times 100\%$$ (2)

Here, *A*_*0*_ represents the absorbance of the blank, and *A*_*1*_ represents the absorbance of the samples.

### 2.12 Antibacterial activity analysis

In this analysis, two representative bacterial strains, namely, the Gram-positive *Staphylococcus aureus* (ATCC 6538) and Gram-negative *Escherichia coli* (ATCC 25922), served as representative bacteria to analyze the *in vitro* antibacterial efficacy of HDCP hydrogels. In this context, HDCP hydrogel disks, measuring 4 mm in diameter and 1.5 mm in thickness, were created and underwent sterilization using UV light. Following this, 100 μL of *E. coli* and *S. aureus* bacterial suspensions with a concentration of 107 U/mL were individually applied to nutrient agar (NA) plates, each having a diameter of 90 mm. Subsequently, the plates were placed in a humidified incubator at 37°C for 24 h with the HDCP hydrogel disks. After incubation, the diameters of the inhibition zones were measured, and each experiment was independently conducted three times.

### 2.13 Biocompatibility evaluation

To evaluate the cytotoxicity of HDCP hydrogels, mouse embryonic fibroblast cells (NIH-3T3 cells, CRL-1658TM, ATCC) were chosen as the model. NIH-3T3 cells (1 × 105/well, 100 μL) were seeded in a 96-well plate with a 1% penicillin-streptomycin solution and 10% fetal bovine serum (FBS) DMEM at 37 °C (5% CO_2_). After subjecting each hydrogel to sterilization through immersion in 75% ethanol under UV for 30 min and subsequent washing with sterilized PBS three times, HDCP hydrogels were introduced into the cell culture. Subsequently, 10 μL of CCK-8 solution was added to each well after 24, 48, and 72 h, followed by an incubation period of 1 h. The data were recorded at 450 nm using a microplate reader. Each experiment was conducted in triplicate. The morphologies of cells after co-culturing with the hydrogel extract for 24 and 72 h were observed using phalloidin-FITC and 4′6-diamidino-2-phenylindole (DAPI) staining through an inverted phase microscope and a laser confocal fluorescence microscope.

### 2.14 Statistical analysis

Each experiment was performed in triplicate, and the data were presented as means accompanied by the standard deviation (±SD). SPSS software (SPSS Inc, Chicago IL) was employed for the analysis. Statistical significance between groups was determined through analysis of variance (ANOVA), with a significance level set at a *p*-value of <0.05, <0.01, and <0.001 for 95%, 99%, and 99.9% confidence, respectively.

## 3 Results and discussions

### 3.1 Synthesis of CS-PBA and HA-DA

**FIGURE 1:**
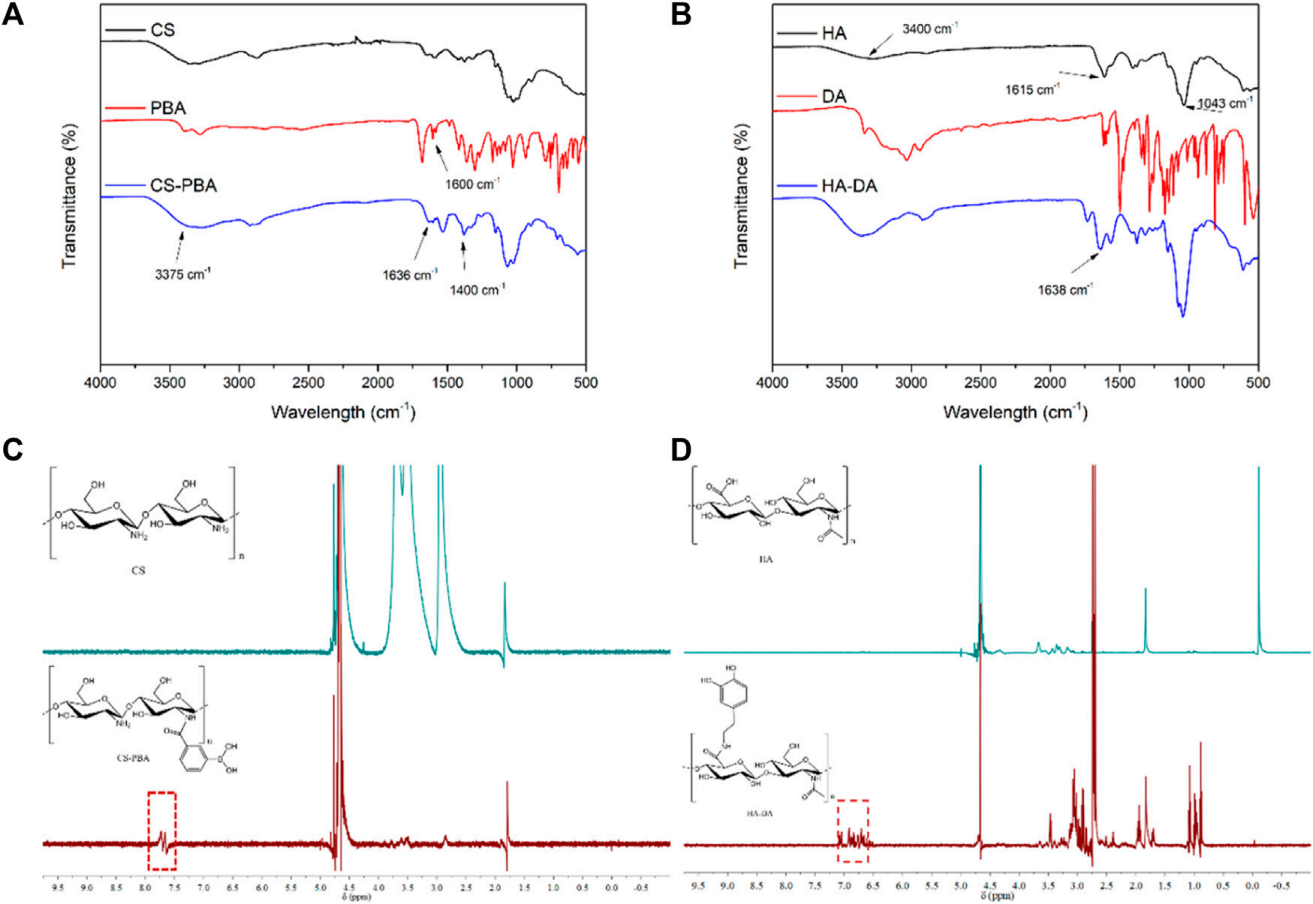
FT-IR spectra of **(A)** CS-PBA and **(B)** HA-DA. ^1^H-NMR spectra of **(C)** CS-PBA and **(D)** HA-DA.

FT-IR and ^1^H NMR analyses were utilized to discern the structure of CS-PBA and HA-DA polymers (refer to Figure 1). In Figure 1A, the distinctive absorption peak at 1,636 cm^−1^ in CS-PBA indicates the presence of the amide bond. The peaks observed at 3,375 cm^−1^ and 1,450–1,600 cm^−1^ correspond to the N-H stretching vibration of the amide bond and the skeleton vibration of the benzene, respectively, signifying the successful modification of CS with phenylboronic acid. Figure 1B provides confirmation of grafted dopamine through an amide reaction in hyaluronic acid. The broad absorption peak around 3,400 cm^−1^ corresponds to the O-H bond, while the robust absorption peaks at 1,043 cm^−1^ and 1,615 cm^−1^ are attributed to the C-O and N-H bonds, respectively. The spectra of HA-DA reveal a pronounced peak at 1,638 cm^−1^, indicating the presence of hyaluronan through amide bonds formed by dopamine grafting, thereby affirming the successful DA grafting onto HA. Furthermore, the emergence of a new peak ranging from 7.5 to 8.0 ppm in Figure 1C suggests the successful grafting of phenylboronic acid onto the chitosan segment. The substitution degree of phenylboronic acid onto the chitosan could be calculated using ^1^H NMR, and in this work, the substitution degree of phenylboronic acid was ∼8%. Additional clarification on the chemical structure of HA-DA was obtained through ^1^H NMR analysis (Figure 1D). The ^1^H NMR spectrum of HA-DA reveals a distinct chemical shift at 6.7 ppm, indicating the presence of the proton peak from the benzene ring in the DA catechol group. According to the ^1^H NMR spectrum of HA-DA, the degree of substitution of dopamine (i.e., catechol conjugation efficiency) was ∼10%. The above results confirmed the successful grafting of dopamine onto hyaluronic acid (HA) through the amide reaction.

### 3.2 Fabrication of HDCP hydrogels

**FIGURE 2:**
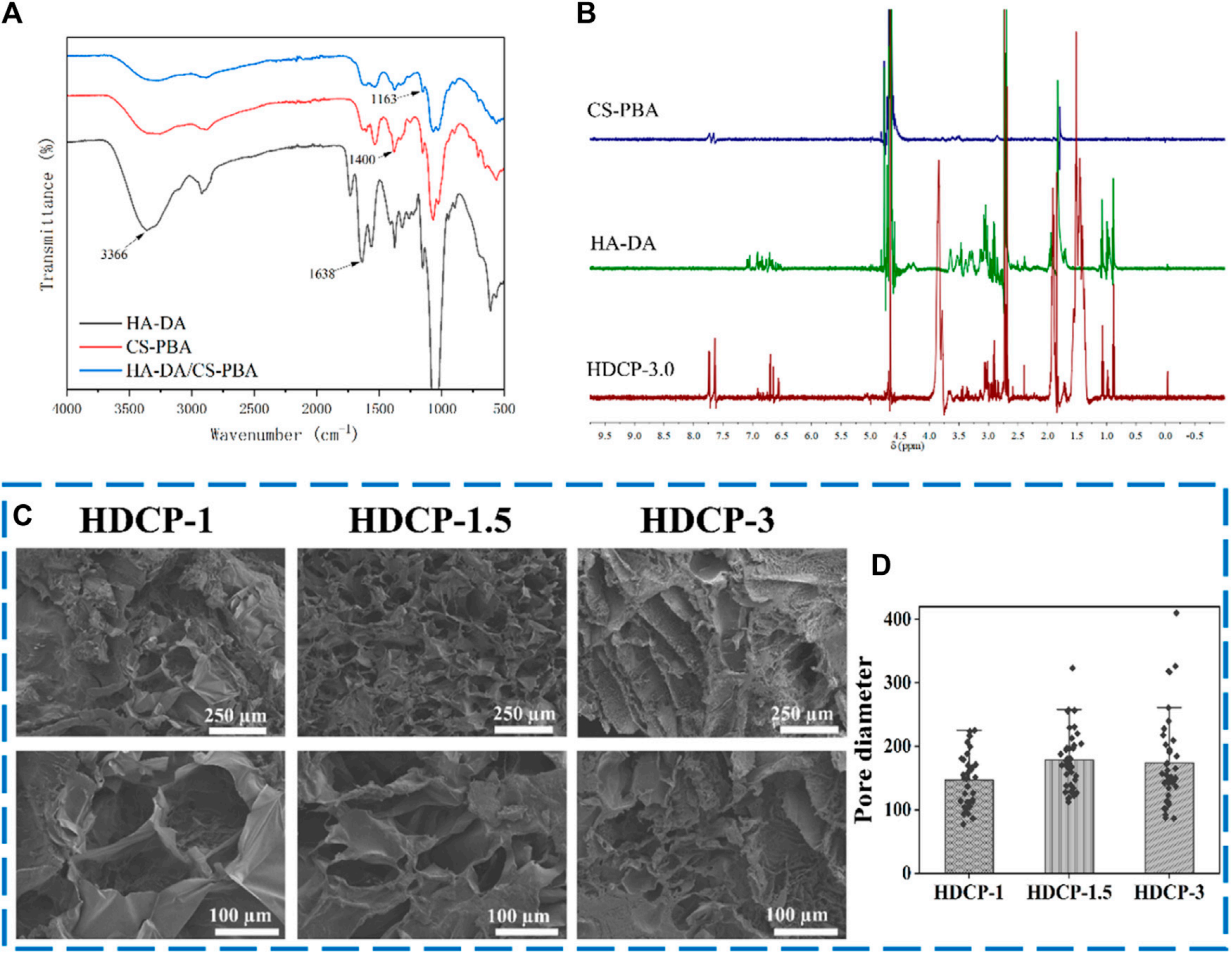
FT-IR spectra **(A)** and ^1^H NMR spectra **(B)** of HDCP hydrogels. **(C)** SEM images of HDCP hydrogels, and **(D)** the pore size distribution analysis of HDCP hydrogels according to SEM images using ImageJ software.

The FT-IR spectrum of HDCP hydrogels, the prominent peak at 1,163 cm^-1^ (see Figure 2A) is due to the borate bond, signifying the positive interaction between CS-PBA and HA-DA polymers. The 1H-NMR spectrum of the HDCP hydrogel further supports the successful crosslinking between the two polymers (see Figure 2B). Subsequently, the morphology and microstructure of the HDCP hydrogels were examined using SEM (see Figure 2C). Porosity is essential for fostering the process of wound healing when hydrogels are used (Liu et al., 2023). It influences not only the rate and depth of cell growth *in vivo*, as well as cell adhesion and activity, but also affects the processes of nutrient and waste exchange, influencing cell permeability. As shown in Figure 2C, all hydrogels display a porous structure, aiding the transport of molecules during the wound healing process. According to the pore size distribution analysis using the software ImageJ according to SEM images (5 SEM images for each sample) (Figure 2D), sample HDCP-1.5 exhibits a higher pore size than the other two samples. SEM images indicate relatively uniform and interconnected pore structures, suggesting good structural stability and a homogeneous chemical composition.

### 3.3 Rheological analysis

**FIGURE 3:**
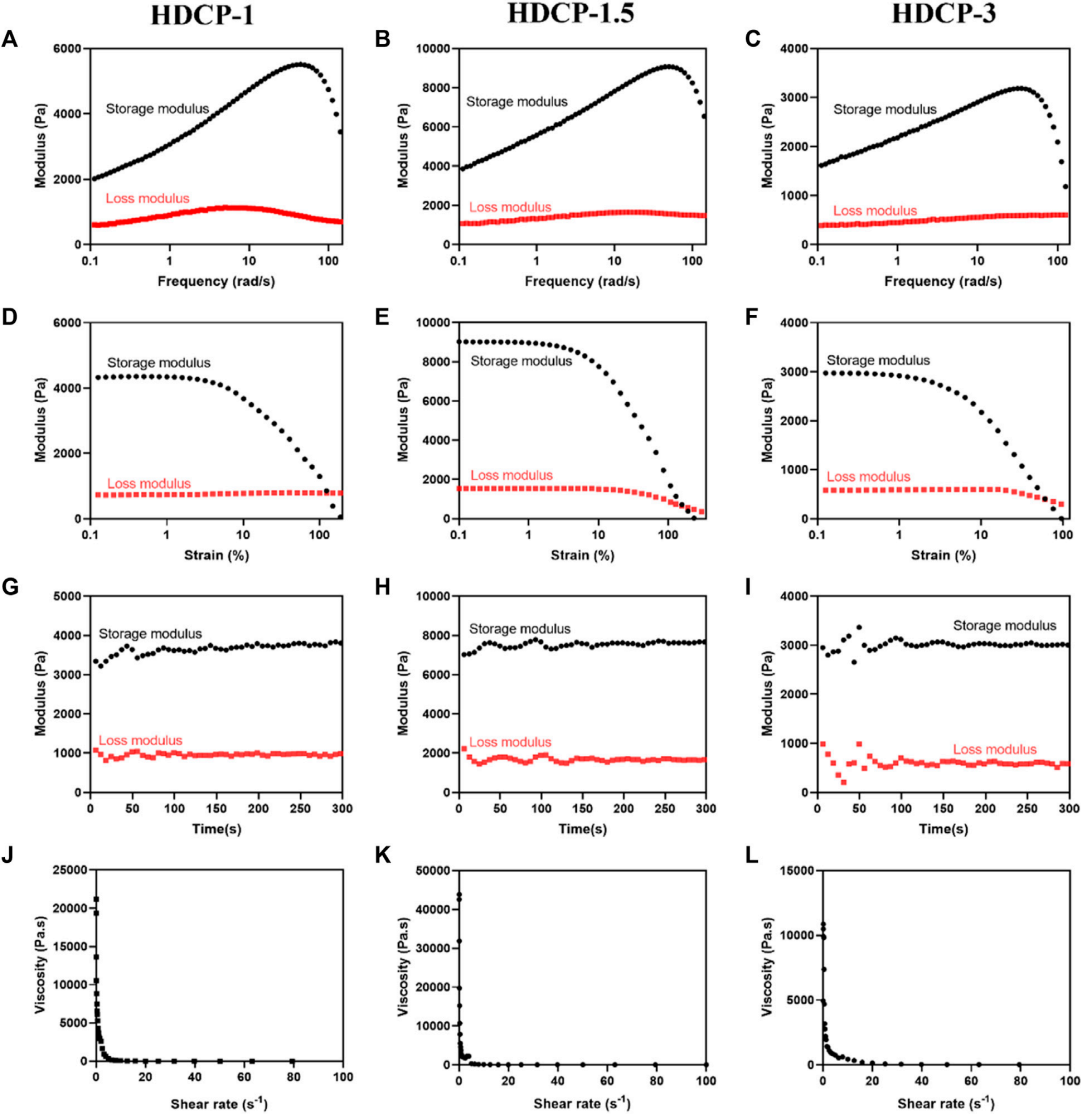
Rheological characteristics of the formulated HDCP hydrogels were assessed, including the storage modulus (G′) and loss modulus (G″), examined across frequency **(A–C)**, strain **(D–F)**, and time **(G–I)**. Alterations in the viscosity of HDCP hydrogels concerning shear rate were also investigated **(J–L)**.

Figure 3 illustrates that all HDCP hydrogels exhibit comparable non-linear rheological characteristics, with the storage modulus (G′) always higher than the corresponding loss modulus (G″) throughout, affirming the elastic solid behavior of the hydrogels (see Figures 3A–C). Additionally, upon the initial application of stress, the hydrogels demonstrate the expected behavior, where the G′ value exceeds the G″ value. Especially, sample HDCP-1.5 displayed higher G′ and G″ values than that of HDCP-1 and HDCP-3 at the first stage of strain (since 0% strain), mainly due to the fact that sample HDCP-1.5 had the optimal ratio of CS-PBA and HA-DA, resulting in the higher crosslinking density than other two samples. However, a prolonged increase in stress can lead to the convergence of G′ and G″, showing the degradation of the network structure within the hydrogel (see Figures 3D–F). The strain tolerance of the hydrogels before “structural failure” follows the order: HDCP-1.5 > HDCP-1 > HDCP-3. Furthermore, the stability of G′ and G″ over time indicates the hydrogels’ consistent behavior, with G′ consistently exceeding G'' (see Figures 3G–I). Furthermore, hydrogels exhibiting excellent injectability can efficiently fill irregular wound areas. Consequently, the injectability of the HDCP hydrogel was further evaluated by analyzing the viscosity’s dependence on the shear rate using a rheometer. Illustrated in Figures 3J–L, the apparent viscosities of the diverse HDCP hydrogels decrease as the shear rate increases, showcasing shear-thinning behavior. In conclusion, the rheological findings suggest that all HDCP hydrogels possess advantageous shear-thinning and injectability characteristics.

### 3.4 Swelling behavior and adhesion behavior

**FIGURE 4:**
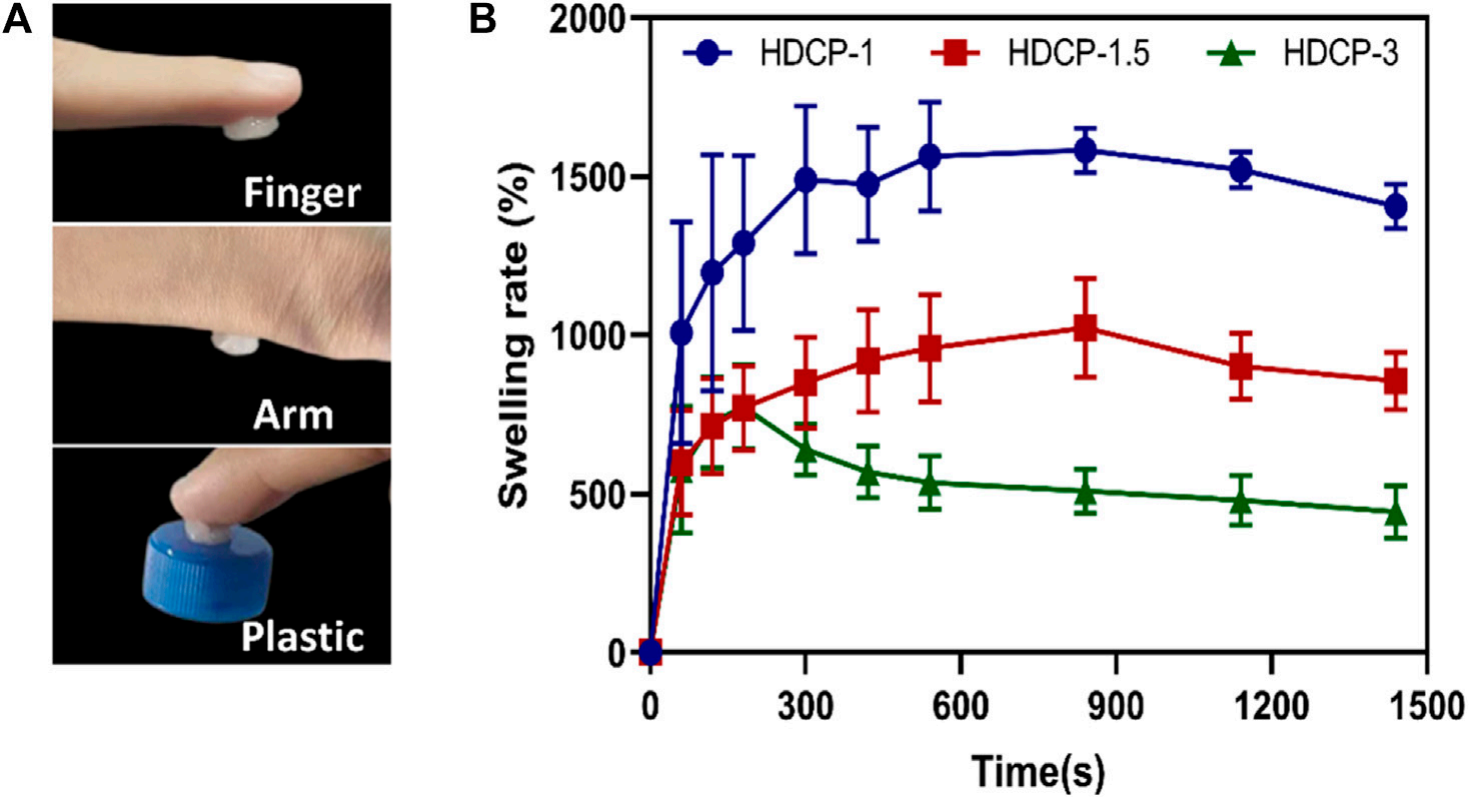
**(A)** Photographs display the HDCP hydrogel (HDCP-1) adhered to the human finger and arm, and plastic. **(B)** Swelling property of HDCP hydrogels in DI water.

Figure 4A shows that the HDCP hydrogels have good adhesiveness on the human arm, finger, and plastic surface. The adhesion was assigned to the interactions between unreacted phenolic hydroxyl groups and the surrounding skin tissue surface, including hydrogen bonds and electrostatics (Nie et al., 2023). Moreover, studies have indicated that the adhesive strength is contingent on the hydrogel’s cohesion and interfacial adhesiveness. The heightened crosslinking density enhances the hydrogel’s cohesion, thereby augmenting its adhesiveness (Liang et al., 2021b). Furthermore, a hydrogel with absorbent capabilities can efficiently soak up excess exudate from the wound area, promoting the wound healing process (Liu et al., 2022). Figure 4B, shows that all HDCP hydrogels possess good swelling properties after soaking in deionized water due to the interconnection network and porous structure of hydrogels. Moreover, HDCP-1 exhibits better swelling behavior compared to HDCP-1.5 and HDCP-3.

### 3.5 Antibacterial properties

**FIGURE 5:**
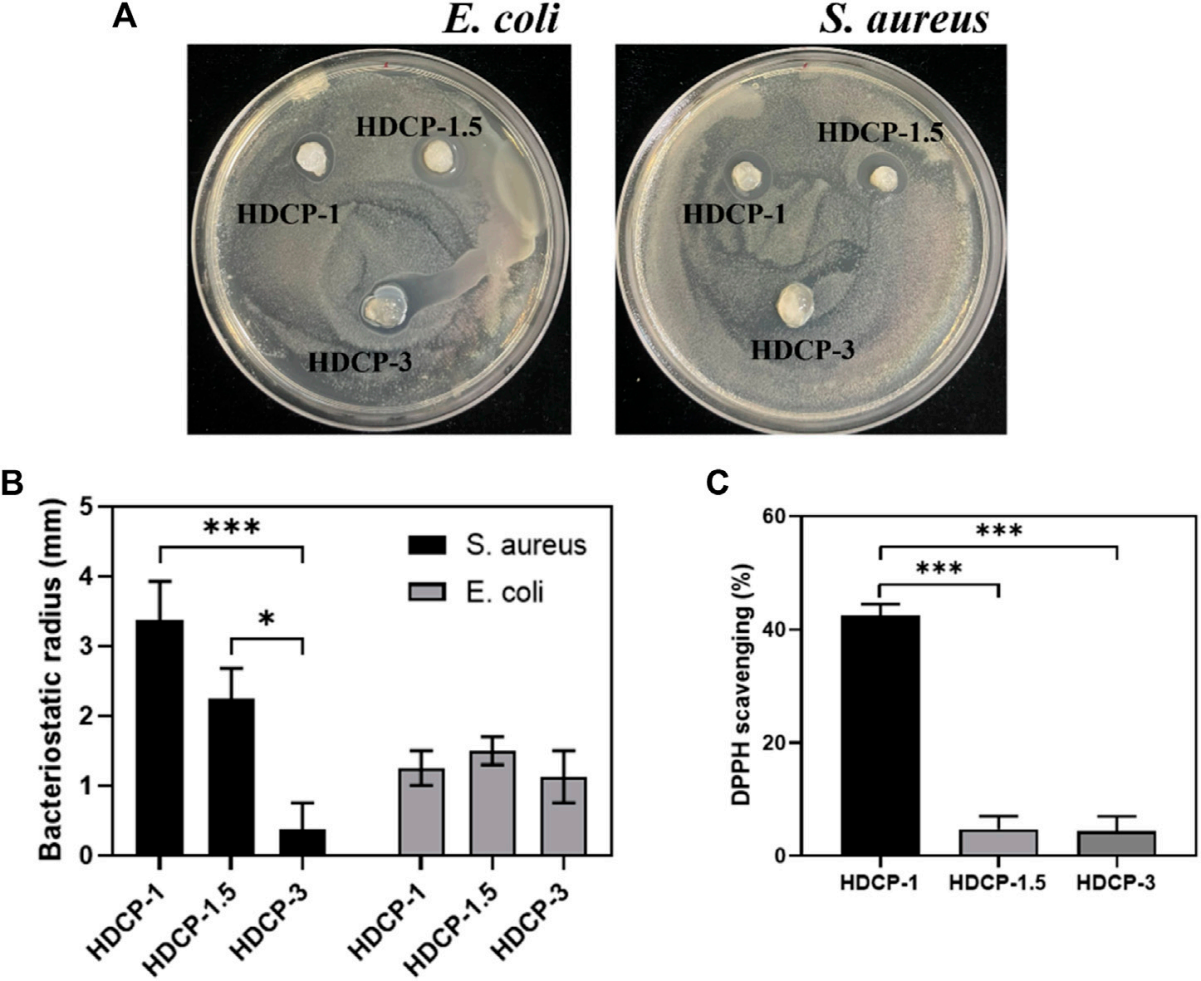
**(A)** Images depicting the antibacterial activity of the hydrogels against *E. coli* and *S. aureus* after 24 h. **(B)** The recorded radius of the inhibitory zone of the hydrogels against *E. coli* and *S. aureus*. **(C)** Assessment of the DPPH scavenging effect of HDCP hydrogels. *p < 0.05, **p < 0.01, and ***p < 0.001.

The composite coatings were subjected to a contact-killing bacteria experiment to evaluate their antibacterial activity. Two bacterial models, Gram-negative *E. coli* and Gram-positive *S. aureus*, were employed. An inhibition zone test was conducted for both *E. coli* and *S. aureus* to assess the antibacterial efficacy of HDCP hydrogels (Figures 5A, B). The results revealed varying degrees of antibacterial effects for all HDCP hydrogels, as depicted in Figure 5B. Notably, HDCP-1 exhibited more consistent and enduring antibacterial properties against *S. aureus* compared to other HDCP hydrogels. On the other hand, HDCP-1.5 displayed the most enduring antibacterial effects against *E. coli* compared to the other hydrogels.

### 3.6 Antioxidant activity

In the dynamic progression of chronic wound healing, inflammation is initiated by infection or cellular exudate, prompting the activation of the immune system and the generation of reactive oxygen species (ROS). The excessive build-up of ROS in cells disrupts the equilibrium between oxidants and antioxidants, resulting in tissue damage, intensified infections, and hindered wound healing. (Hamidi et al., 2022). Hence, it is crucial to promptly and continuously eliminate excess ROS from the damaged tissue to facilitate effective wound healing. As depicted in Figure 5C, all HDCP hydrogels demonstrated a certain degree of clearance of DPPH, with HDCP-1 hydrogel achieving ∼42% DPPH clearance rate. This occurrence can be attributed to the transfer of electrons or the provision of hydrogen atoms from nitrogen ion segments to DPPH free radicals.

### 3.7 *In vitro* cytocompatibility evaluation

**FIGURE 6:**
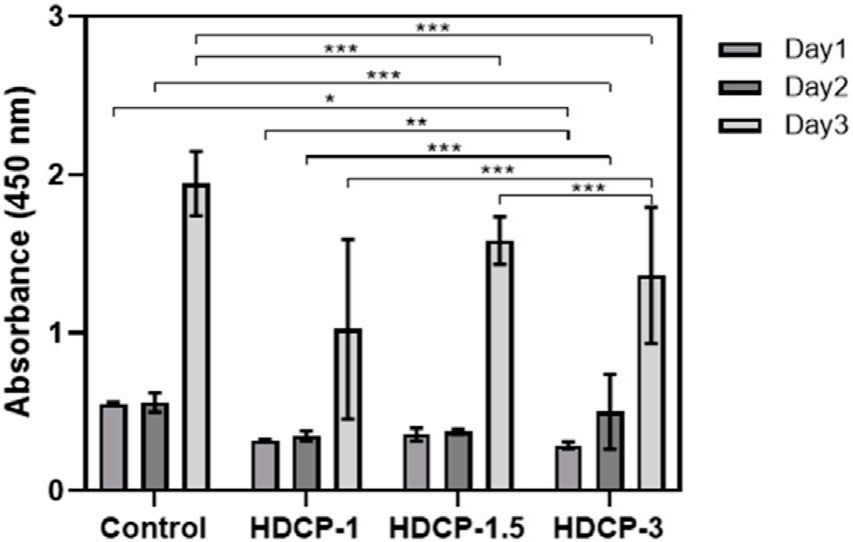
The cytocompatibility of HDCP hydrogels was evaluated through CCK-8 after incubation with NIH-3T3 cells for various days. The absorbance at 450 nm was measured, and a control group without the addition of hydrogels was included. *p < 0.05, **p < 0.01, and ***p < 0.001.

**FIGURE 7:**
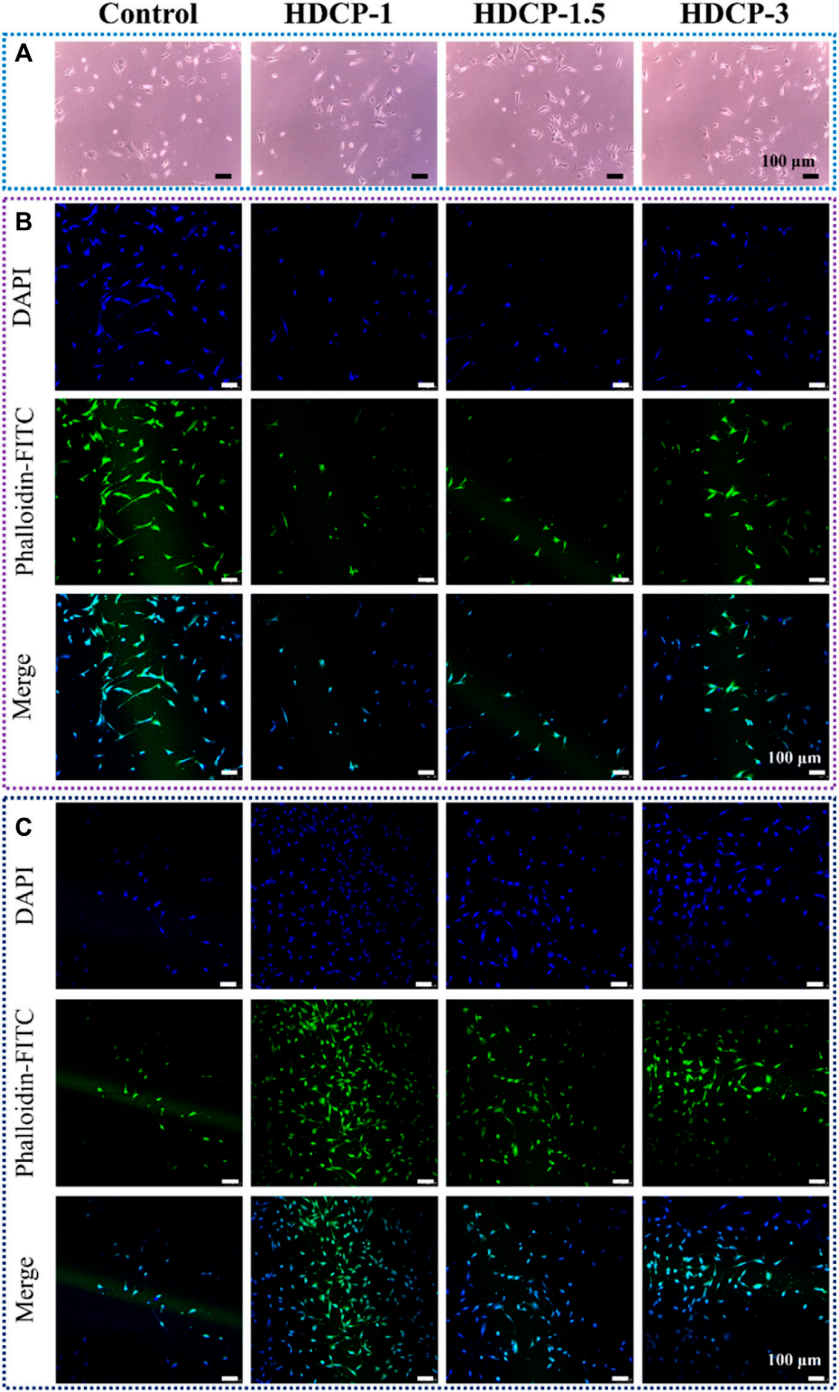
The assessment of biocompatibility for HDCP hydrogels included: **(A)** Optical microscope images capturing NIH-3T3 cells cultured with HDCP hydrogel extracts on day 1. **(B)** Fluorescent microscopy images displaying NIH-3T3 cells cultured with HDCP hydrogel extracts for 1 day, stained with phalloidin-FITC/DAPI. **(C)** Corresponding images for 3 days of cell culture.

The cytocompatibility of HDCP hydrogels was initially evaluated through the standard CCK-8 method, involving the cultivation of NIH-3T3 cells for varying durations, as illustrated in Figure 6. Based on the CCK-8 results, the proliferation of NIH-3T3 cells showed an upward trend across all HDCP hydrogels from day 1 to day 3, with a notably substantial increase noticed in the HDCP-1.5 hydrogel. These outcomes suggest cytocompatibility of HDCP hydrogels. Furthermore, the evaluation of NIH-3T3 cells cultured with extracts from HDCP hydrogels on both day 1 and day 3 involved examination using an inverted phase microscope and a laser confocal fluorescence microscope (Figure 7). The images reveal that after 24 h of co-culture with the hydrogel extracts at 37 °C, NIH-3T3 cells exhibited normal morphology (Figure 7A), and the cell count increased with prolonged cultivation time (Figures 7B, C), indicating the hydrogels possess cytocompatibility.

## 4 Conclusion

This research showcases innovative HDCP hydrogels produced by utilizing dopamine-modified hyaluronic acid (HA-DA) and phenylboronic acid-modified chitosan (CS-PBA), interconnected through boric acid ester bonds crosslinking. The investigation extended to exploring the physicochemical, antibacterial, and cytocompatibility features of the HDCP hydrogels. The resulting hydrogels exhibited a porous structure along with favorable rheological, adhesive, and swelling properties. Additionally, the HDCP hydrogels displayed significant antioxidant activity, effective antibacterial characteristics, and good cytocompatibility. These findings collectively underscore the promising potential of HDCP hydrogels across various biomedical applications, particularly in the realm of wound dressing.

## Data Availability

The raw data supporting the conclusion of this article will be made available by the authors, without undue reservation.
